# The impact of parental substance use disorder and other family-related problems on school related outcomes

**DOI:** 10.1016/j.dadr.2022.100041

**Published:** 2022-03-16

**Authors:** Kirsten Søndergaard Frederiksen, Morten Hesse, Julie Brummer, Mads Uffe Pedersen

**Affiliations:** Centre for Alcohol and Drug Research, Aarhus University, Bartholins Allé 10, Building 1322, 218, Aarhus C DK-8000, Denmark

**Keywords:** Parental substance use disorder, School performance, Types of family-related problems, Register data, Survey data, Latent class analysis

## Abstract

**Aims:**

To identify young people with different levels of family-related problems, including parental substance use disorder (PSUD), and investigate differences in grades at graduation from compulsory school and further enrollment in education.

**Methods:**

Participants included 6784 emerging adults (aged 15–25 years) from samples drawn for two national surveys in Denmark 2014-2015. Latent classes were constructed using the following parental variables: PSUD, offspring not living with both parents, and parental criminality, mental disorders, chronic diseases and long-term unemployment. The characteristics were analyzed using an independent one-way ANOVA. Differences in grade point average and further enrollment were analyzed using linear regression and logistic regression, respectively.

**Results:**

Four classes of families were identified: 1. “Low adverse childhood experiences (ACE) families”, 2. “Families with PSUD”, 3. “Families with unemployment” and 4. “High ACE families”. There were significant differences in grades, with the highest average among youth from “Low ACE families” (males = 6.83; females = 7.40) and significant lower averages among both males and females from the other types of families, but lowest among young people from “High ACE families” (Males = 5.58; females = 5.79). Youth from “Families with PSUD” (Males: OR = 1.51; 95% CI: 1.01-2.26; females: OR = 2.16; 95% CI: 1.22-3.85) and “High ACE families” (Males: OR = 1.78; 95% CI: 1.11-2.26) were significantly more likely not to be enrolled in further education compared with “Low ACE families”.

**Conclusions:**

Young people who experience PSUD, both as the primary family-related problem as well as among multiple family-related problems, are at increased risk for negative school-related outcomes.

## Highlights

• Four types of families with different levels of family-related problems were identified.
• Grades at graduation were lower for children with more family-related problems.
• Children from two types of families were more likely not to continue education.
• The two types had parental substance use disorder + few/many other problems.

## 1 Introduction

How do family-related problems affect children's success in school and their educational attainment? School is perhaps the most pivotal context outside the home and where children spend a significant amount of time. Success in school and academic achievements have an effect on the later health, well-being and problem behavior of individuals (Gauffin et al., 2013; Hawkins et al., 1992; Herke et al., 2020; Johnson and Leff, 1999). At a societal level, governments often focus on educational attainment as a tool to promote social mobility (Landersø and Heckman, 2017), and researchers have argued (and debated) how having a well-educated general population is economically beneficial (Browne et al., 2010; Hanushek, 2016). School is often seen as a catalyst for changes in families (Chilton et al., 2015), or at least as an institution with possibilities for reducing social inequalities and increasing social mobility (Iannelli, 2013).

This points to the importance of looking into the impact of adverse family background for success or failure in the educational system. Children enter the school environment and navigate through the school years with varying levels of family-related problems and adverse childhood experiences (ACEs), including parental substance use disorders (PSUD), parental mental disorders, parental early death, neglect and domestic violence. Research has shown how different kinds of ACEs can impact not only family life but also children's relations to other children and adults, later mental health, and substance use (Bellis et al., 2015; Björkenstam et al., 2017; Dovran et al., 2019; Dunn et al., 2013; Kessler et al., 2010). The severity, level and number of ACEs also correlate with school hardship and learning difficulties (Dovran et al., 2019), schooling attainment (Cawley et al., 2001), as well as lack of school engagement, school absenteeism and repeating grades (Crouch et al., 2019; Robles et al., 2019).

PSUD is of particular interest, as it is a potentially modifiable ACE. Children living with PSUD experience more challenges in the school setting compared with their peers (Sher, 1997) and are at greater risk for low academic performance, skipping school days and dropping out of school (Berg et al., 2016b; Casas-Gil and Navarro-Guzman, 2002a; Chandy et al., 1993; Hafekost et al., 2017; Knop et al., 1985; Murphy, 1991).

Even though the relationship between PSUD and children's school performance has been quite comprehensively researched, there are significant limitations in the existing literature. PSUD does not exist in a vacuum and is often linked to other family-related problems that may influence a child's upbringing adversely (Ellis et al., 1997; Hafekost et al., 2017). Socioeconomic status (SES) is one factor that has been shown to be associated with both educational achievement and substance use disorders (Kendler et al., 2020; von Stumm et al., 2020). However, the link between SES and substance use disorders is likely to differ between countries and regions (Grittner et al., 2020), and in the Danish context, the correlation between heavy drinking and SES is very weak (Bloomfield et al., 2006). Other family-related problems have only sporadically been taken into account in the research on school performance. A full analysis of school performance, where different parental mental, somatic, legal and work-related problems are included, would be a significant contribution to the literature. The failure to take other family-related problems into account may explain some of the inconsistencies in the existing research Casas-Gil and Navarro-Guzman (2002b). included a control group in their study about school performance and parental alcohol use disorder but did not include other family-related problems in the analysis Hafekost et al. (2017). investigated maternal alcohol use disorder and school attendance but did not have data about the family environment to include in the analysis, and, on this basis, the underlying reasons for non-attendance were difficult to ascertain Berg et al. (2016a). did include information about parental crime and mental health problems. However, the study focused on parental hospital admissions for alcohol-related disorders and, thus, primarily captured only the most severely affected families. This resulted in less variation in the levels of problems, but, most importantly, it reflected another limitation in the existing research. Much of the previous research is based solely on clinical populations (parents in alcohol or drug treatment or children in family services) or families otherwise identified with multiple problems (Casas-Gil and Navarro-Guzman, 2002a; Khemiri et al., 2020; Knop et al., 1985; Miller and Jang, 1977; Murphy, 1991) and makes it difficult to generalize the findings to a broader context that includes children from different layers of society with different levels of family problems. This highlights the importance of including populations across a range of levels of problems. Lastly, some of the research relies on one type of data source only, like registers, or data from the parents, such as clinical interviews or questionnaires. Self-reports from children on PSUD provide insight into problems not defined externally by researchers but by the emerging adults themselves. By combining self-reports with rich longitudinal register-data, it is possible to capture a wider range of families.

### 1.1 Present study

By including a range of family-related problems and different levels of problem severity, the present study addresses some of the shortcomings in previous research. The primary aim of the study was to compare grades at graduation from compulsory school and further enrollment in education after compulsory school among Danish 15–25-year-olds from different types of families with or without PSUD and other family-related problems. We hypothesized that children experiencing PSUD would have lower grades and be less likely to continue their education and that additional stressful events and family-related problems would compound the negative impact of PSUD.

## 2 Materials and methods

The analyses drew on data from two national sample surveys among 15–25-year-old Danish young people (the National YouthMap Surveys), which were conducted by the center for Alcohol and Drug Research in 2014 and 2015 (Pedersen et al., 2017). By combining these cross-sectional datasets with register data on both the young adults and their parents, it was possible to study school performance and family-related problems from the birth of the young adults through their adolescence.

### 2.1 The survey data

The National YouthMap Surveys investigated substance use, wellbeing, and different kinds of social, psychological and physical problems. Of the 10,414 young people invited to take part in the surveys, 5755 participated (55.3%). Details on the National YouthMap Surveys, the methods and design have been published elsewhere (Frederiksen et al., 2021; Pedersen et al., 2018, 2017).

### 2.2 The register-based data

The population and health care registers in Denmark contain individual-level data on the entire population and can be linked to survey data through a personal identification number (Thygesen et al., 2011). We used a family relation register to identify the parents of the young people invited to participate in the National YouthMap Survey. Register data on grades from the General Certificate of Secondary Education as well as register data on further enrollment in education were then extracted and linked with the survey data on a secure server at Statistics Denmark.

Other registers were used to identify the following parental and family-related problems during the young adults’ childhood: parental criminality, parental mental disorders, parental chronic diseases, parental long-term unemployment, and separation from parents (Table 1). Registrations were identified in the period from the young person's birth until their 15th birthday, except parental mental disorders and chronic diseases, which were tracked until the time of the survey, as diagnosis for these types of conditions can often be delayed (Cornally and McCarthy, 2011; Green et al., 2020; Oleski et al., 2010; Scott and Walter, 2010).

**Table 1:** Information selected from population-based registers.

Register	Measure	Years
The Student Register	Grades from the General Certificate of Compulsory Education	2001–2016
The Employment Classification Module	Further enrollment in education	1985–2018
The National Patient Register	Parental chronic diseases	1989–2015
The Psychiatric Central Research Register	Parental mental disorder diagnoses (except alcohol- and drug-related disorders)	1989–2015
The Danish Central Crime Register	Parental convictions/charges (except some types of traffic offenses)	1989–2015
Danish registers on personal labor market affiliation	Parental long-term unemployment	1989–2015

### 2.3 The study population

**Fig. 1:**
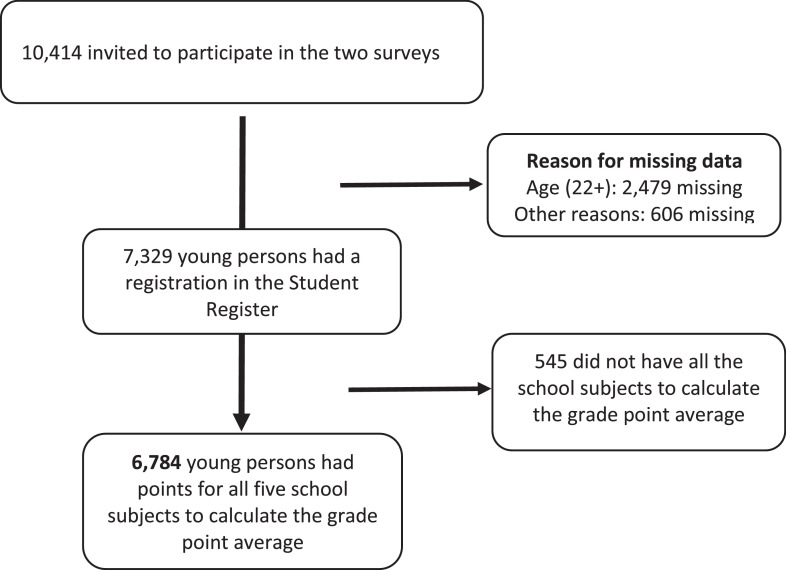
Flowchart for study population.

The study population was derived from the random sample of 10,414 young adults invited to participate in the two surveys. The present study included a subgroup of 6784 young people who had an entry in the school register and a grade point average calculated on the basis of the official guidelines (for school subject guidelines and weights, see Table 3; for the study population, see the flowchart in Fig. 1).

Of the sample of 10,414 young people, 3630 had missing values in the Student Register. Participants with missing school data and the study population differed in the distribution of sex (53.3% vs. 50.5% male), ethnicity (73.5% vs. 91.3% with Danish origin) and parents’ highest level of education (32.4% vs. 7.8% with compulsory education, 42.6% vs. 52.4% with upper secondary and 25.0% vs. 39.8% with higher education) (Table 2). One reason for the missing values was the implementation timeline of the register, which was introduced in 2001 but did not have full coverage until 2006. Other reasons were that participants may have been sick or institutionalized, or may have attended schools that do not hold exams (such as Waldorf Schools that opt out of exams). Lastly, immigrants were more likely to have missing data, perhaps because they had been living elsewhere during the time they graduated.

**Table 2:** Comparison of the group with missing data in the Student register and the study population.

		Missing Student Register data *n* = 3630	Study population*n* = 6784	Overall sample*N* = 10,414	Chi2Pr = 0.007
Sex					
	Male	53.3%	50.5%	51.4%	
	Female	46.7%	59.5%	48.6%	
Ethnicity					<0.001
	Danish origin	73.5%	91.3%	85.1%	
	Descendants/ immigrants	26.5%	8.7%	14.9%	
Parents’ highest level of education					<0.001
	CompulsoryUpper secondaryHigher education	32.4%42.6%25.0%	7.8%52.4%39.8%	16.3%49.0%34.7%	

### 2.4 Measures concerning grades at graduation and further enrollment in education

#### 2.4.1 Grades from the general certificate of compulsory education

Compulsory education in Denmark spans nine years, from approximately age 6 to 15. Most young people attend public school, and these students participate in national exams. Most private schools do the same, with the exception of schools such as Waldorf Schools that opt out of exams. Information on the participants’ grade point averages was obtained from the Student Register covering the years 2001–2016 (Sørensen, 2020). The grade point system in Denmark is a scale from −3 to 12 (−3, 0, 2, 4, 7, 10 and 12), and higher grades indicate better performance. Grades of −3 and 0 are failing marks, 7 is the general average and 12 indicates complete fulfillment of the goals of the subject matter. A continuous variable with the weighted grade point average was calculated on the basis of the official guidelines (The Ministry of Children and Education, 2020) (Table 3). The grades were thus based on exams taken when the participants were approximately 15 to 16 years old.

**Table 3:** Official guidelines for grade point average.

Compulsory exams	Weighting
Danish, oral	100%
English, oral	100%
Physics/chemistry biology and geography, oral	100%
Mathematics, written	50%
Danish, written:	
Orthography	25%
Reading	25%
Written representation	50%

#### 2.4.3 Further enrollment in education

Further enrollment in education was defined as any registration in the category of “Enrolled in education” in the Employment Classification Module (Petersson et al., 2011) in the two years following the final examination in school. The education could either be general or vocational upper secondary education. The general upper secondary educations are divided into four different types of preparatory programmes for tertiary education, which are usually for young people ages 15–19 (Education MOCA, 2021). A vocational program is a practical educational program, which qualifies for employment as a skilled worker.

### 2.5 Measures concerning family-related problems

#### 2.5.1 Parental substance use disorder (PSUD)

PSUD was identified using survey and register data. A parent was considered to have PSUD if at least one of the following criteria was satisfied: (1) the young person responded in the survey that their parent had a current or previous substance abuse problem or (2) the parent had a register entry for a substance-related disease, disorder, charge/conviction, cause of death or treatment (for further information, see Frederiksen et al. 2021).

By combining self-report and register-based measures, it was, on the one hand, possible to get information on the families that did not appear in the register, and, on the other hand, it was possible to get information from the registers on the non-participants of the survey studies (Frederiksen et al., 2021).

PSUD was reported by 447 of the young adults, and 947 of the young adults had parental registrations for substance-related contacts. Some of the young persons both reported PSUD in the survey and had parental registrations for PSUD. With the combination of self-reports and the information from the registers, a total of 1145 (16.9%) young adults had PSUD. This measure of PSUD included young adults with PSUD reported in the survey alone, PSUD reported in the survey and identified in the register, and PSUD identified in the register alone.

#### 2.5.2 Parental long-term unemployment

Information on parents’ employment status was obtained from Danish registers on personal labor market affiliation (Petersson et al., 2011). Long-term unemployment was defined as three consecutive years, or more than three non-consecutive years, of social benefit receipt or unemployment (including unemployment benefits and early retirement but not State Education Support or parental leave).

#### 2.5.3 Not living with both parents

Information was obtained about whether or not the young person lived with both biological parents from the year of birth up to and including their 15th birthday. If the child lived apart from one or both parents during one or more years, they were considered to have experienced “Not living with both parents”.

#### 2.5.4 Parental chronic, serious physical disease

A parent was classified as having a chronic, serious disease if they had received a primary or secondary diagnosis in the National Patient Register (Lynge et al., 2011) for the following diseases (Sundhedsdatastyrelsen, 2017; World Health Organization, 2004): type 2 diabetes, chronic obstructive pulmonary disease, asthma, rheumatoid arthritis and osteoporosis (International Classification of Diseases (ICD-10) codes: E11, J44, J45, M05, M06, M80, M81 and M82). The National Patient Register contains records for all hospital contacts in Denmark, including inpatient, outpatient, and acute contacts.

#### 2.5.5 Parental mental health problems

A parent was considered to have had a mental disorder if they had any record in the Psychiatric Central Register (Mors et al., 2011; Sahl Andersen et al., 2011) (except F10-F19 diagnoses, which were included in the PSUD measure). Similar to the National Patient Register, the Psychiatric Central Register contains all types of hospital-based episodes of psychiatric care, including inpatient, outpatient and acute episodes.

#### 2.5.6 Parental criminality

Parental criminality was defined as a conviction or charge registered in the Danish Central Crime Register (Ravn, 2001)*.* Convictions and charges related to traffic offenses were excluded. Driving under the influence was included as an indicator of PSUD.

### 2.6 Ethics

Participants in both surveys were informed of the purpose of the survey in an invitation letter in which the voluntary participation and confidentiality measures were detailed. Participants indicated their informed consent by completing the survey. Both studies, which involved the linking of the register and the survey data, were registered at the Danish Data Protection Agency, and all confidentiality and privacy requirements were met.

### 2.7 Statistical methods of analysis

A Latent Class Analysis (LCA) was performed to investigate different types of families with different levels of family-related problems, including PSUD. LCA is a powerful and flexible method for identifying and understanding unobserved groups in a population. Based on the existing research, we believed that there were groups among the young people with different family-related problems and that these groups would have different school outcomes. Using LCA, we fitted a model to determine which individuals were likely to belong to each group based on information available from the survey and register data. The following variables were included in the LCA model:

■ -/+ parental mental disorder
■ -/+ parental criminality
■ -/+ parental long-term unemployment
■ -/+ not living with both parents
■ -/+ parental chronic disease
■ -/+ PSUD

One, two, three, four and five classes were fitted using multinomial logistic regression models and compared to determine which of these models fits best. We used goodness-of-fit statistics, Akaike's and Bayesian information criterion (AIC and BIC), entropy and the classification probabilities to determine the best fit (Table 4).

**Table 4:** Model fit information.

Number of classes	BIC	AIC	Entropy	Predicted posterior probability	LRTp-value
1	38,770	38,729	.	1	0.000
2	36,449	36,361	.69	.91	0.000
3	36,335	36,199	.36	.78	0.000
**4**	**36,309**	**36,132**	**.63**	**.80**	**0.039**
5	36,331	36,113	.65	.82	0.86

LRT: Likelihood ratio test; AIC: Akaike's information criterion; BIC: Bayesian information criterion

Note: The selected model with four classes is marked with bold type.

After the LCA was performed, a descriptive analysis used an independent one-way ANOVA to investigate differences in the characteristics of the young persons from the classes. Differences between the latent classes in terms of grade point average and further enrollment in education were analyzed using linear regression and logistic regression, respectively. Both models controlled for ethnicity (Danish origin vs. immigrant/descendants) and parents’ level of education (compulsory education only vs. any higher education). We tested for interaction between the classes and offspring sex. Due to the significant interactions, we stratified both models by sex (male/female). The analyses were run with STATA 15 and 16, as well as R (R Core Team, 2013; StataCorp., 2019).

## 3 Results

The comparison showed the model with four classes had the lowest BIC (see Table 4). Based on the latest recommendations on using BIC (and not AIC or the chi-square test), as well as considerations about the theoretical meaningfulness of the classes, the model with four classes was selected as the best-fitting model for our data (Nylund et al., 2007; Schreiber, 2017; Weller et al., 2020).

**Fig. 2:**
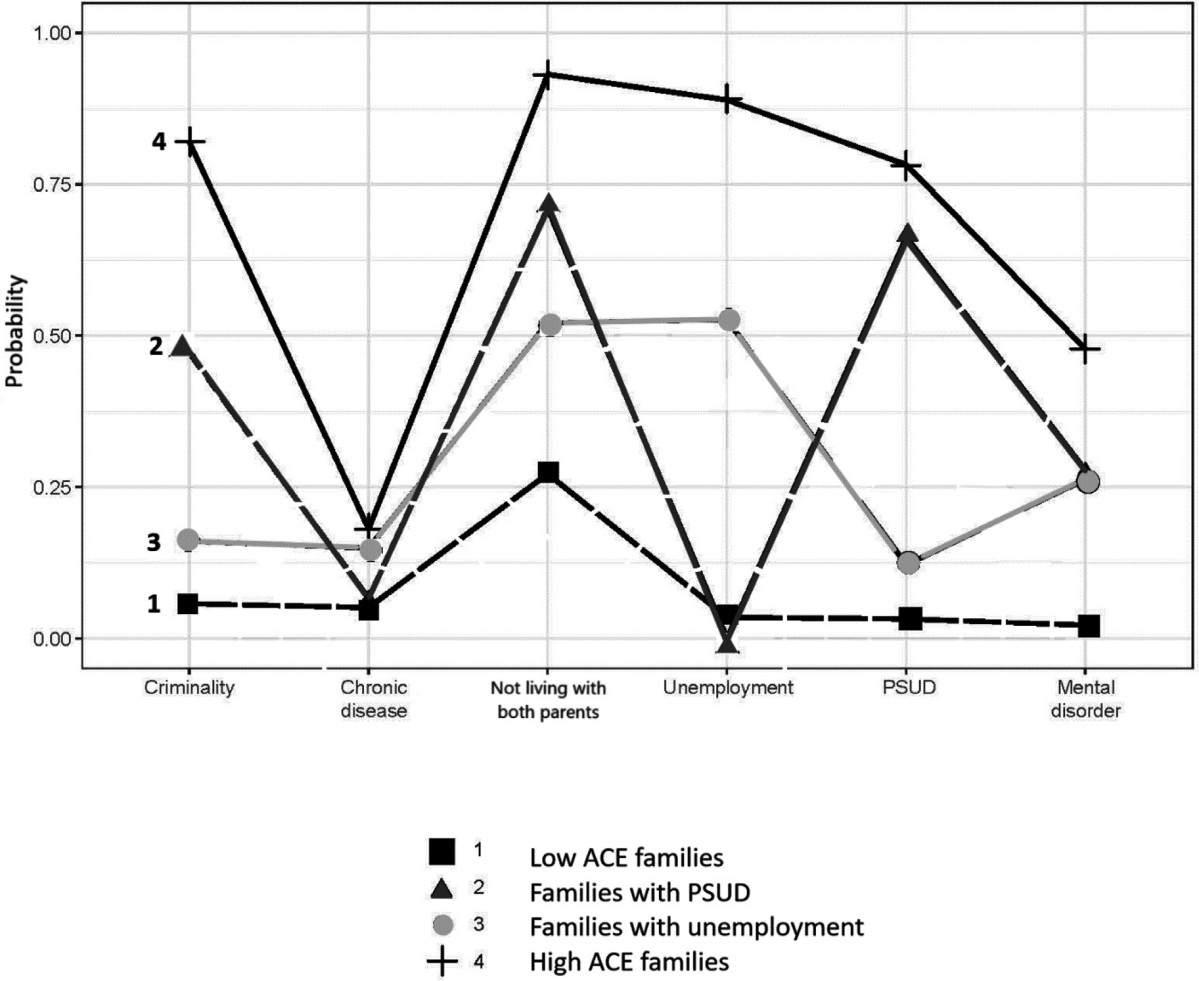
Marginal probabilities for the four classes of having six family-related problems.

Based on the four-class model, the identified classes were labelled 1. “Low ACE families” (*n* = 4351; 64%), 2. “Families with PSUD” (*n* = 549; 8%), 3. “Families with unemployment” (*n* = 1477; 22%) and 4. “High ACE families” (*n* = 407; 6%). The latent class marginal means (Fig. 2) showed the probability in each group of having one or more of the family-related problems.

For “Low ACE families” (Class 1), the marginal probabilities were low for all the different family-related problems. For “Families with PSUD” (Class 2), the marginal probabilities were high for PSUD (0.66) and not living with both parents (0.71) and, to a lesser extent, for parental criminality (0.47). The marginal probability was lower for parental mental disorders (0.28) and particularly low for parental chronic diseases (0.07) and parental long-term unemployment (<0.001).

“Families with unemployment” (Class 3) had high marginal probabilities of not living with both parents (0.52) and parental long-term unemployment (0.53). The marginal probability of parental mental disorders was not high but still present for many of the families (0.28). The marginal probability of parental chronic diseases was quite low (0.15) but higher than for “Low ACE families” and “Families with PSUD” (Class 1 and Class 2, respectively). Lastly, “High ACE families” (Class 4) showed the highest marginal probabilities in all areas: PSUD (0.78), not living with both parents (0.93), parental criminality (0.82), parental long-term unemployment (0.89), parental mental disorders (0.48) and parental chronic diseases (0.18).

As shown in Table 5, the young people from the four types of families differed especially with regard to ethnicity (F(3,6780)=241.9, *p*<0.001) and parental level of education (F(3,6780)=107.5, *p*<0.001). In terms of ethnicity, the post hoc Bonferroni test revealed no differences between “Low ACE families” and “Families with PSUD” or between “Families with unemployment” and “High ACE families” but significant differences between the other types of families. In terms of parental education, the post hoc Bonferroni test showed significant differences between all four classes. Offspring sex differed between some of the types of families (F(3,6780)=4.81, *p* = 0.002), and the post hoc Bonferroni test revealed differences between “Low ACE families” and “High ACE families” as well as between “Families with unemployment” and “High ACE families”.

**Table 5:** Descriptive characteristics from a one-way independent ANOVA for young people from the four types of families (*N* = 6784).

	Low ACE families*n* = 4351	Families with PSUD*n* = 549	Families with unemployment*n* = 1477	High ACE families*n* = 407	P-value
Offspring sex					0.002
Males	2207 (50.7%)	259 (47.2%)	781 (52.9%)	176 (43.2%)	
Females	2144 (49.3%)	290 (52.8%)	696 (47.1%)	231 (56.8%)	
Offspring ethnicity					<0.001
Danish	4208 (96.7%)	530 (96.5%)	1131 (76.6%)	322 (79.1%)	
Immigrants/descendants	143 (3.3%)	19 (3.5%)	346 (23.4%)	85 (20.9%)	
Parents with low level of education (compulsory education only)	172 (4.0%)	49 (8.9%)	224 (15.2%)	88 (21.6%)	<0.001

### 3.1 Level of family-related problems and school performance

**Fig. 3:**
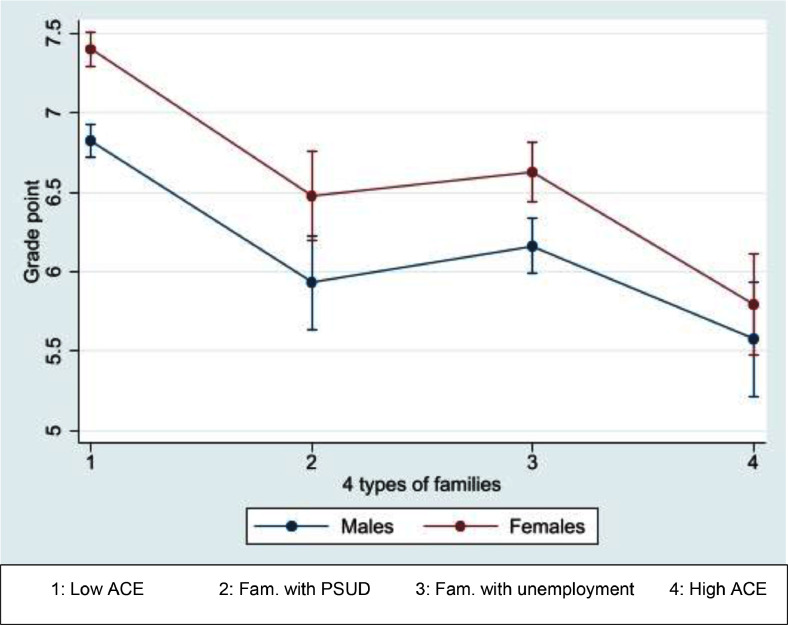
Predictive margins (incl. 95% confidence intervals (CI)) for the young people's grade point averages by family type and offspring sex.

The grade point average (Fig. 3) was highest among young persons, both males and females, from “Low ACE families” (grade point average for males: 6.83, 95% CI: 6.72–6.93; for females: 7.40, 95% CI: 7.30–7.50). Young persons from “High ACE Families” had the lowest grade point average, and young persons from “Families with PSUD” had almost the same grade point average as youth from “Families with unemployment”. Females had significantly higher grade point average compared with males in all families, except those from “High ACE families” (5.79; 95% CI: 5.48–6.11). Among males, the lowest grade point average was observed among those from “High ACE families” (5.58; 95% CI: 5.22–5.94) and “Families with PSUD” (5.93; 95% CI: 5.64–6.23) with overlapping confidence intervals.

Of the 6784 young people, only 420 (6.2%) were not enrolled in some kind of education program after graduation (Table 6). Using young people from “Low ACE families” as the reference group, higher odds of not being enrolled in education were observed for young people from “Families with PSUD” and “High ACE families”, in particular among females (“Families with PSUD”: OR=2.16, 95% CI: 1.22–3.85; “High ACE families”: OR=3.41, 95% CI: 1.96–5.93). Females, but not males, from “Families with unemployment” had higher odds of not being enrolled in further education (OR=2.08; 95% CI: 1.32–3.28).

**Table 6:** No further enrollment in education, by the four latent classes of young people stratified by offspring sex a (*N* = 6778 b) from logistic regression.

	Low ACE families(*n* = 4347)	Families with PSUD(*n* = 549)	Families with unemployment(*n* = 1475)	High ACE families(*n* = 407)
Not enrolled	237 (5.5%)	48 (8.7%)	90 (6.1%)	45 (11.1%)
Males	Ref.	OR = 1.51*p* = 0.04495% CI: 1.01–2.26	OR = 0.91*p* = 0.55495% CI: 1.01–2.26	OR = 1.78*p* = 0.01695% CI: 1.11–2.26
Females	Ref	OR = 2.16*p* = 0.00995% CI: 1.22–3.85	OR = 2.08*p* = 0.00295% CI: 1.32–3.28	OR = 3.41*p* < 0.00195% CI: 1.96–5.93

a Controlled for ethnicity and parents’ level of education.

b 6 missing.

## 4 Discussion

Consistent with our hypothesis, PSUD and other concurrent family-related problems had an impact on the young people's school performance and chance of being further enrolled in education after completing compulsory school. The analysis indicated that different types of family-related problems have different levels of impact.

The results of the present study are consistent with prior research showing that experiencing higher levels of adversity in childhood is associated with poorer school-related outcomes. In a study of a population with a high risk for reported adversities (adult participants recruited in prisons and substance abuse and mental health treatment settings), severity levels were strongly associated with the likelihood of school difficulties and hardship at school (Dovran et al., 2019). As well, a general population cross-sectional study concluded that negative school outcomes were associated with a higher ACE score and lower levels of protective factors (Robles et al., 2019). At the same time, parts of the analysis from the present study show a less clear-cut relationship between family-related problems and negative school outcomes, as youth from “Families with long-term unemployment” had higher probabilities of experiencing different family-related problems but did not have a higher risk of not being enrolled in further education. In comparison with “Low ACE families”, this family type had higher probabilities of parental criminality, chronic diseases and mental disorders, as well as parents living separately, and, thus, the burden of problems in the families was potentially quite comprehensive. But, these young people were not at greater risk of dropping out of the educational system. This finding indicates that different types of family-related problems have different impacts on school performance and that PSUD may be an important factor with regard to the well-being of young people. Previous research on the impact of PSUD versus other ACEs has produced mixed results. Some studies have suggested that a dysfunctional family structure has the greatest impact on the well-being of young people irrespective of PSUD (Anda et al., 2002; Christoffersen and Soothill, 2003), while other studies have concluded that PSUD has independent effects (Jääskeläinen, 2016; Rognmo et al., 2012).

In the present study, the two family types that included PSUD (“Families with PSUD” and “High ACE families”) had significantly lower school grade averages and a higher risk of no further enrollment compared with “Low ACE families”, and the differences were more distinct between females from the four family types with regard to the latter outcome. Compared with males, females had significantly better school outcomes across the different types of families, except in “High ACE families”, which had equally poor school outcomes for both females and males.

### 4.1 Strengths and limitations of the study

A major strength of this study was the combination of self-reports and register data, which allowed us to look at young people's school outcomes and parental problems not only at the time of the survey but also from the children's birth through their adolescence and early adulthood. Registers capture families, parents and young adults with more severe problems that are often rarer and more difficult to cover with survey studies (Brummer et al., 2021). However, register data are limited to those who use the services or receive some kind of benefit or punishment (Jääskeläinen, 2016). By also using survey data, the present study captured a more general group of families and parents who do not receive services but still have alcohol or drug problems. Differences between survey participants and non-participants in the distribution of social, mental and psychological problems (Christensen et al., 2015; Groves, 2006) can lead to bias and a potential underestimation of PSUD, but using register data on both participants and non-participants reduces this bias (Frederiksen et al., 2021). Combining register data and self-reports provides a more nuanced understanding of school performance among children with PSUD and other family-related problems.

A limitation in the present study's construction of family-related problems is that we do not know the extent of contact between the parent and child. For example, a parent could have a mental disorder and be very affected by it, but if contact with the child is very sparse, it may not have much impact on the child's life. However, parental problems may affect the child even when the parent is absent (Carbonneau et al., 1998). Another limitation is the lack of data on the timespan of PSUD and when it occurred in the young people's lives (Frederiksen et al., 2021). Furthermore, the retrospective nature of the self-reports together with the historical register data do not allow us to draw any conclusions about a causal effect of PSUD on school performance. Rather, the present study shows how PSUD and additional family-related problems compound the negative impact on school performance.

In addition, we had missing register data on several subsets of participants. Immigrants were more likely to have missing data than Danish participants, which may reflect that they had been living elsewhere during the time they graduated and thus would not have had data entered in the database. Other reasons for missing data could be that some young people attended schools that do not hold exams, and some young people may have been sick or institutionalized at the time of the exams.

### 4.2 Implications

This study demonstrates how PSUD and family-related problems are associated with poorer school outcomes. School attendance and success in school are important on many levels and have a major influence on the physical, psychological and social development of children and adolescents (Herke et al., 2020). But if school performance and retention in the educational system are impacted by family-related problems, including PSUD, this can lead to more inequalities, not only for the individual but also on a group level. Research has shown that PSUD can have an adverse impact during the formative years (Christoffersen and Soothill, 2003). The health and well-being of youth can be affected by structural factors, such as national wealth, income inequality and access to education (Viner et al., 2012). At the same time, research has also underscored the importance of protective factors, such as safe and supportive families, peers and schools. Protective factors in school can be positive school experiences, attendance at school, achievement and acknowledgement of success (Velleman and Templeton, 2016). The present study points to a need for a focus, not only in the schools but also in families and social welfare institutions, on protective factors in the social environment for young people who are in families with PSUD and additional family-related problems.

## 5 Conclusions

The present study investigated how a range of family-related problems, in particular PSUD, had an impact on young people's grades at graduation from compulsory school and further enrollment in education. Four groups of young people with varying levels of family-related problems were identified. The results of this study show how young people who experience PSUD, both as the primary family-related problem as well as among multiple family-related problems, are at increased risk for negative school-related outcomes.

## Author disclosures

### Role of funding source

The funding source for the present study is the Danish National Budget. The Danish Government had no say in the decision to publish or the content of the present manuscript.

## Contributors

All authors are from the same university and the same department. All authors have participated in the research and/or article preparation. All authors have approved the final article.

## CRediT authorship contribution statement

**Kirsten Søndergaard Frederiksen:** Conceptualization, Data curation, Formal analysis, Methodology, Software, Writing – original draft, Writing – review & editing. **Morten Hesse:** Conceptualization, Formal analysis, Writing – review & editing. **Julie Brummer:** Conceptualization, Formal analysis, Writing – review & editing. **Mads Uffe Pedersen:** Conceptualization, Funding acquisition, Investigation, Project administration, Resources, Supervision, Writing – review & editing.

## Declaration of Competing Interest

None.

## Appendix Supplementary materials

Image, application 1

Image, application 2
