# Supplementary material for: The impact of parental substance use disorder and other family-related problems on school related outcomes
Source: Drug Alcohol Depend Rep. 2022 Mar 16;3:100041. doi: 10.1016/j.dadr.2022.100041 (PMC9948819; doi:10.1016/j.dadr.2022.100041)
Supplement: Supplementary file 1 [file mmc1.docx]

Table: Latent class marginal means

|  | Number of obs = 6,784 |  | Delta- | method |  |  |
| --- | --- | --- | --- | --- | --- | --- |
|  |  | Pred. prob, N | Margin | Str. Err | 95 % | CI |
| Low ACE |  | 4,351/6,784  (64%) |  |  |  |  |
|  | PSUD | 144/4,351 | .032 | .009 | .018 | .055 |
|  | Mental disorders | 0/4,351 | .022 | .011 | .008 | .058 |
|  | Chronic disease | 254/4,351 | .051 | .006 | .041 | .064 |
|  | Not living with both parents | 1,339/4,351 | .271 | .013 | .246 | .299 |
|  | Criminality | 352/4,351 | .057 | .007 | .045 | .072 |
|  | Long-term unemployment | 0/4,351 | .035 | .022 | .010 | .114 |
| PSUD |  | 549/6,784  (8%) |  |  |  |  |
|  | PSUD | 506/549 | .662 | .081 | .490 | .800 |
|  | Mental disorders | 198/549 | .278 | .051 | .190 | .387 |
|  | Chronic disease | 37/549 | .066 | .022 | .034 | .124 |
|  | Not living with both parents | 445/549 | .710 | .062 | .575 | .815 |
|  | Criminality | 295/549 | .471 | .071 | .337 | .610 |
|  | Long-term unemployment | 0/549 | -18 | . | . | . |
| Long-term unemployment |  | 1,477/6,784  (22%) |  |  |  |  |
|  | PSUD | 144/1,477 | .121 | .026 | .079 | .181 |
|  | Mental disorders | 535/1,477 | .262 | .031 | .206 | .328 |
|  | Chronic disease | 232/1,477 | .149 | .017 | .119 | .184 |
|  | Not living with both parents | 738/1,477 | .521 | .037 | .449 | .592 |
|  | Criminality | 202/1,477 | .160 | .027 | .114 | .220 |
|  | Long-term unemployment | 1,143/1,477 | .526 | .074 | .383 | .665 |
| High ACE |  | 407/6,784  (6%) |  |  |  |  |
|  | PSUD | 351/407 | .782 | .044 | .685 | .856 |
|  | Mental disorders | 242/407 | .477 | .029 | .422 | .534 |
|  | Chronic disease | 76/407 | .181 | .022 | .142 | .226 |
|  | Not living with both parents | 378/407 | .931 | .021 | .877 | .963 |
|  | Criminality | 367/407 | .820 | .040 | .727 | .886 |
|  | Long-term unemployment | 402/407 | .889 | .117 | .441 | .988 |
