# Supplementary material for: The impact of parental substance use disorder and other family-related problems on school related outcomes
Source: Drug Alcohol Depend Rep. 2022 Mar 16;3:100041. doi: 10.1016/j.dadr.2022.100041 (PMC9948819; doi:10.1016/j.dadr.2022.100041)
Supplement: Supplementary file 2 [file mmc2.docx]

**Title: The impact of parental substance use disorder and other family-related problems on school related outcomes**

**Authors:**

Kirsten Søndergaard Frederiksen (0000-0001-8092-530X),^1^

Morten Hesse (0000-0002-6849-6554),^1^

Julie Brummer (0000-0002-4811-7430)

Mads Uffe Pedersen (0000-0003-1964-1062)^1^

^1^Centre for Alcohol and Drug Research, Aarhus University

**Corresponding author:**

Kirsten Søndergaard Frederiksen

Centre for Alcohol and Drug Research, Aarhus University

Bartholins Allé 10, Building 1322, 218

DK-8000 Aarhus C

Email: kf.crf@psy.au.dk

**Word count:**

3854

**Declarations of interest**

None.
